# Supplementary material for: Using arterial–venous analysis to characterize cancer metabolic consumption in patients
Source: Nat Commun. 2020 Jun 23;11:3169. doi: 10.1038/s41467-020-16810-8 (PMC7311411; doi:10.1038/s41467-020-16810-8)
Supplement: Supplementary file 1 — Supplementary Information [file 41467_2020_16810_MOESM1_ESM.pdf]

Using arterial-venous analysis to characterize cancer metabolic consumption in patients

Xiong *et al.*

Supplementary Figure 1

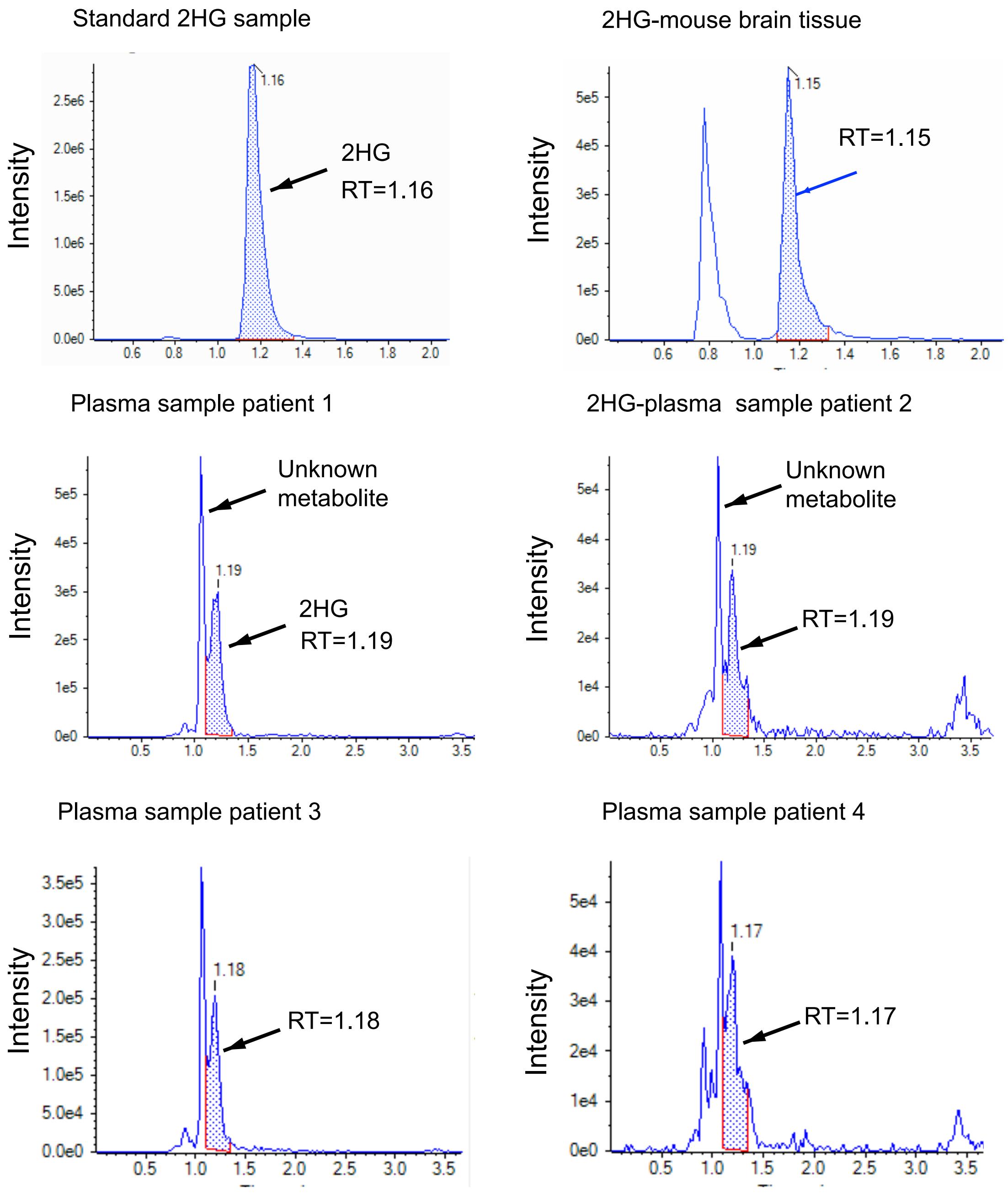

Signals of 2HG from different samples.
Signals of 2HG from standard 2HG samples, extraction from mouse brain tissues and human blood plasma. Signal of 2HG from our measurement with QTRAP5500 (AB SCIEX QTRAP 5500 LC/triple quadrupole mass spectrometer) is always fused with the peak of another unknown metabolite in plasma. They had very close retention times. 2HG, 2-hydroxyglutarate.

Supplementary Figure 2

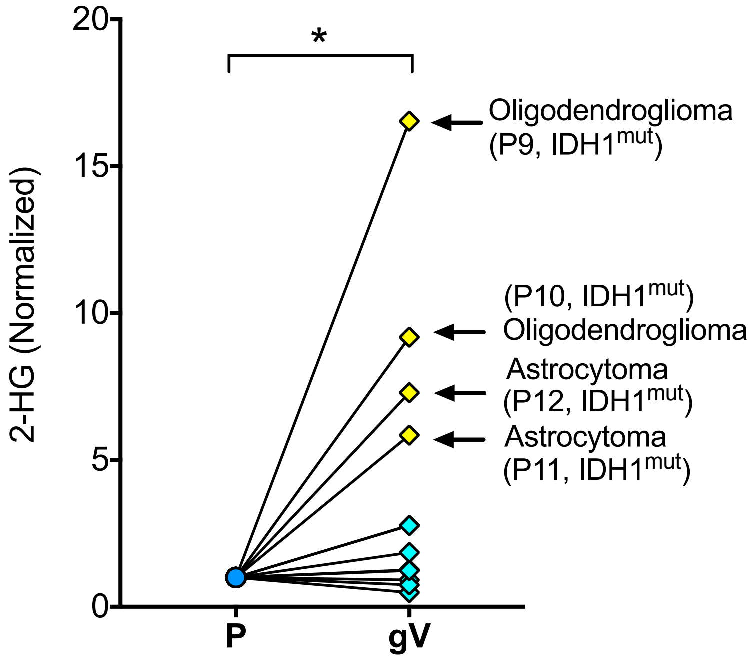

Measurement of 2HG from human blood plasma with QTOF.
The 2HG concentrations in glioma venous samples from patients with grade II and III gliomas (4 of 6 patients, oligodendroglioma and astrocytoma) were much higher than those in other gliomas (e.g., GBM) after their values were normalized to the peripheral vein samples from the same patients. P, samples from pedal vein, gV, samples from glioma veins. *, $p < 0.05$. Two-tailed paired t-test. 2HG, 2-hydroxyglutarate. Source data are provided as a Source Data file.

Supplementary Figure 3

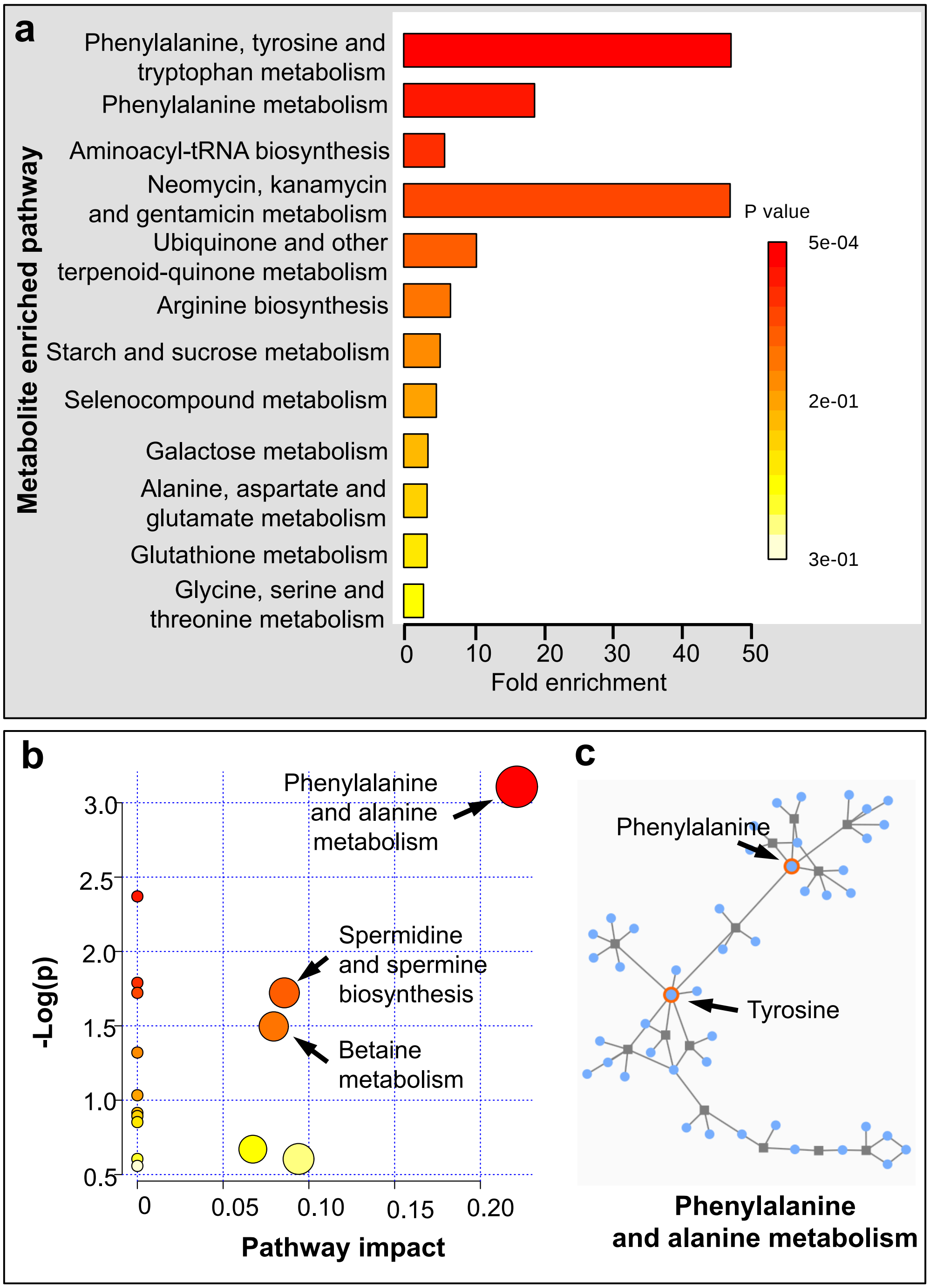

Pathway analysis of arterial enriched metabolites.
Enrichment and impact of arterial plasma enriched metabolites in Figure 2C and 2D (socre >1) were included in the analysis. (**a, b**) The enrichment scores are shown on y-axis, which was calculated as the negative logarithm of the *P*-value from an enrichment test. The x-axis indicates the structural impact with a score from 0 to 1 of the metabolites that are high in arterial plasma compared with those in venous plasma in the enriched pathways. (**c**) Two metabolites (phenylanaline and tyrosine) from phenylanaline, and alanine metabolism enriched in arterial plasma were shown in the pathway.

Supplementary Figure 4

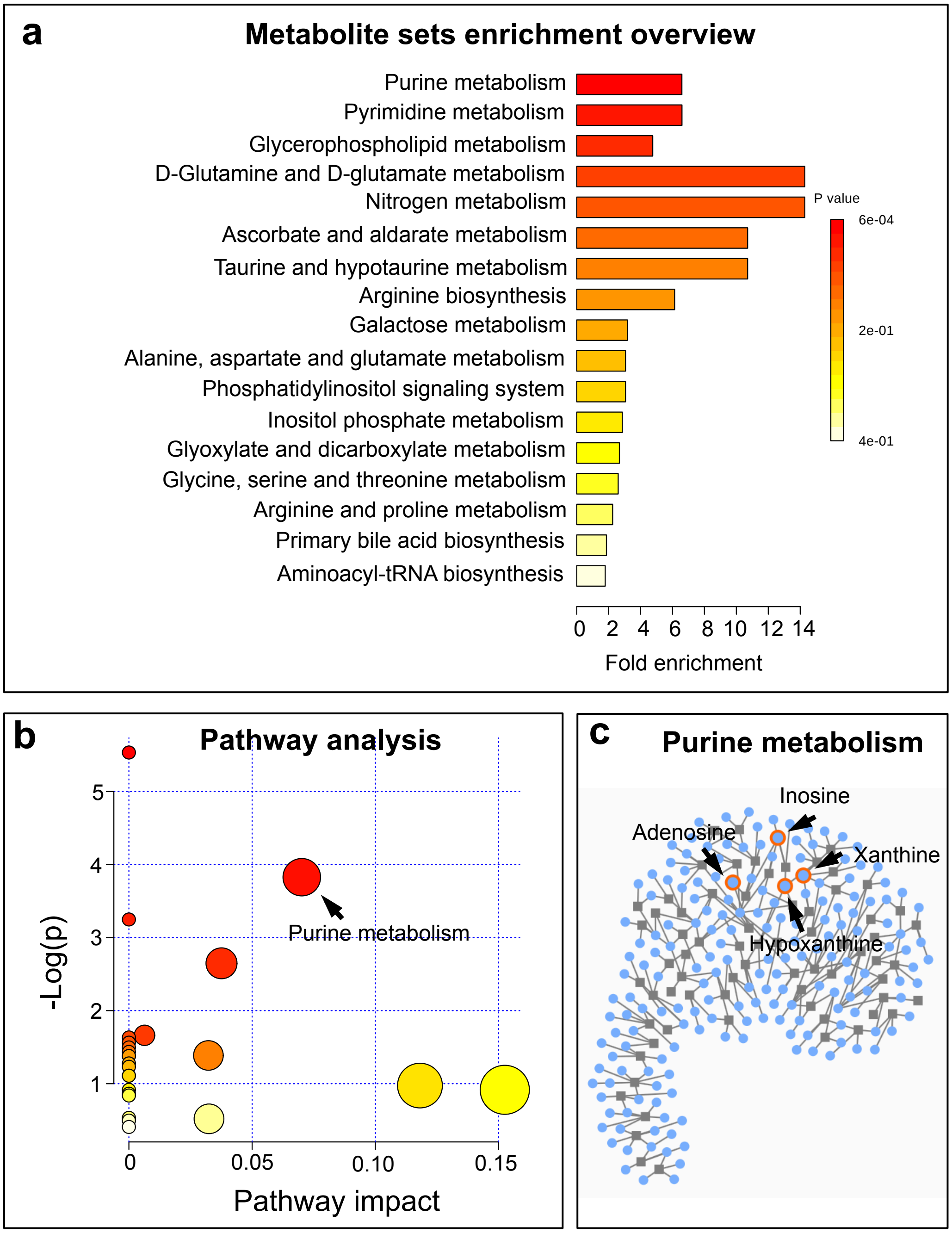

Pathway analyses of vein plasma enriched metabolites.
Enrichment and impact of venous plasma enriched metabolites in Figure 2C and 2D (socre >1) were included in the analysis. (**a, b**)The enrichment scores are shown on y-axis, which was calculated as the negative logarithm of the *P*-value from an enrichment test. The x-axis indicates the structural impact with a score from 0 to 1 of the metabolites, which are high in venous plasma compared with those in arterial plasma in the enriched pathways. (**c**) Four metabolites from purine metabolism pathway enriched in venous plasma were shown in the pathway.

Supplementary Table 1

The information related to patients and brain tumors.

Patient No.	Glioma type	IDH1	ATRX	P53	WHO type
1	Astrocytoma	-	-	-	II
2	Glioblastoma	-	n.a.	n.a.	IV
3	Astrocytoma	n.a.	n.a.	n.a.	II
4	Astrocytoma	n.a.	n.a.	n.a.	III
5	Glioblastoma	-	-	-	III-IV
6	Gliosarcoma	n.a.	n.a.	n.a.	IV
7	Glioblastoma	n.a.	n.a.	n.a.	IV
8	Glioblastoma	n.a.	n.a.	n.a.	III-IV
9	Oligodendroglioma	+	+	+	III
10	Oligodendroglioma	+	+	-	III
11	Astrocytoma	+	-	-	II
12	Astrocytoma	+	-	+	II
13	Glioblastoma	n.a.	n.a.	n.a.	IV

(Age, 21–61 years old, average 48.9±3.7 years old, 46% Male)
