# Supplementary material for: Using arterial–venous analysis to characterize cancer metabolic consumption in patients
Source: Nat Commun. 2020 Jun 23;11:3169. doi: 10.1038/s41467-020-16810-8 (PMC7311411; doi:10.1038/s41467-020-16810-8)
Supplement: Supplementary file 3 — Reporting Summary [file 41467_2020_16810_MOESM3_ESM.pdf]

nature research

Corresponding author(s): Woo-ping Ge
Last updated by author(s): 4/25/2020

Reporting Summary

Nature Research wishes to improve the reproducibility of the work that we publish. This form provides structure for consistency and transparency in reporting. For further information on Nature Research policies, see Authors & Referees and the Editorial Policy Checklist .

Statistics

For all statistical analyses, confirm that the following items are present in the figure legend, table legend, main text, or Methods section.

n/a	Confirmed	
☐	☒	The exact sample size (*n*) for each experimental group/condition, given as a discrete number and unit of measurement
☐	☒	A statement on whether measurements were taken from distinct samples or whether the same sample was measured repeatedly
☐	☒	The statistical test(s) used AND whether they are one- or two-sided<br>*Only common tests should be described solely by name; describe more complex techniques in the Methods section.*
☐	☒	A description of all covariates tested
☒	☐	A description of any assumptions or corrections, such as tests of normality and adjustment for multiple comparisons
☐	☒	A full description of the statistical parameters including central tendency (e.g. means) or other basic estimates (e.g. regression coefficient) AND variation (e.g. standard deviation) or associated estimates of uncertainty (e.g. confidence intervals)
☐	☒	For null hypothesis testing, the test statistic (e.g. *F*, *t*, *r*) with confidence intervals, effect sizes, degrees of freedom and *P* value noted<br>*Give P values as exact values whenever suitable.*
☒	☐	For Bayesian analysis, information on the choice of priors and Markov chain Monte Carlo settings
☒	☐	For hierarchical and complex designs, identification of the appropriate level for tests and full reporting of outcomes
☒	☐	Estimates of effect sizes (e.g. Cohen's *d*, Pearson's *r*), indicating how they were calculated

Our web collection on statistics for biologists contains articles on many of the points above.

Software and code

Policy information about availability of computer code

Data collection	MRM data were acquired using Analyst 1.6.3 software (SCIEX)
Data analysis	MetaboAnalyst4.0, GraphPad Prism 7.0

For manuscripts utilizing custom algorithms or software that are central to the research but not yet described in published literature, software must be made available to editors/reviewers. We strongly encourage code deposition in a community repository (e.g. GitHub). See the Nature Research guidelines for submitting code & software for further information.

Data

Policy information about availability of data

All manuscripts must include a data availability statement. This statement should provide the following information, where applicable:

- Accession codes, unique identifiers, or web links for publicly available datasets
- A list of figures that have associated raw data
- A description of any restrictions on data availability

The data for Figure 2-5, supplementary Figure 2 are provided as source data. The other data are available from the corresponding authors upon reasonable request.

Field-specific reporting

Please select the one below that is the best fit for your research. If you are not sure, read the appropriate sections before making your selection.

☒ Life sciences ☐ Behavioural & social sciences ☐ Ecological, evolutionary & environmental sciences

For a reference copy of the document with all sections, see nature.com/documents/nr-reporting-summary-flat.pdf

October 2018

Life sciences study design

All studies must disclose on these points even when the disclosure is negative.

Sample size	Sample size was determined based on expected effect size and variability within the sample, as well as cost and feasibility of experiments.
Data exclusions	Samples from patients with hemolysis were excluded in this study. These samples contained contamination from blood cells.
Replication	All experiments were replicated at least twice.
Randomization	Samples were randomly assigned to each group by simple randomization.
Blinding	Blinding tests were not feasible in this study.

Reporting for specific materials, systems and methods

We require information from authors about some types of materials, experimental systems and methods used in many studies. Here, indicate whether each material, system or method listed is relevant to your study. If you are not sure if a list item applies to your research, read the appropriate section before selecting a response.

Materials & experimental systems

n/a	Involved in the study	
☐	☒	Antibodies
☒	☐	Eukaryotic cell lines
☒	☐	Palaeontology
☒	☐	Animals and other organisms
☐	☒	Human research participants
☒	☐	Clinical data

Methods

n/a	Involved in the study	
☒	☐	ChIP-seq
☒	☐	Flow cytometry
☐	☒	MRI-based neuroimaging

Antibodies

Antibodies used	IDH1 R132H (clone H09, ZM0447, ZSGB-Bio), ATRX (ZA-0016, polyclonal, ZSGB-Bio) , P53 (DO-7, mouse monoclonal, Roche)
Validation	Validated by manufacturers for the application used.

Human research participants

Policy information about studies involving human research participants

Population characteristics	Human blood samples were from the patients of gliomas or control subjects.
Recruitment	Blood sample were obtained at the time of surgery and collected before removing glioma from patients . Samples from control subjects were obtained from patients without brain tumors.
Ethics oversight	Union Hospital, Tongji Medical College, Huazhong University of Science and Technology (IRB, 2017-S229)

Note that full information on the approval of the study protocol must also be provided in the manuscript.

Magnetic resonance imaging

Experimental design

Design type	No functional MRI (fMRI) data were acquired.
Design specifications	This is not an fMRI study
Behavioral performance measures	This is not an fMRI study

Acquisition

Imaging type(s)	Only structural MRI sequences was used in this study
Field strength	3T
Sequence & imaging parameters	Sequence:a clinical 3.0T scanner (Magnetom Verio, Siemens Healthcare, Erlangen, Germany) equipped with a 12-channel head coil using T1-weighted coronal and axial imaging and T2- weighted/fluid attenuated inversion recovery (FLAIR) axial imaging. Postcontrast T1-weighted images were acquired after injection of either gadopentetate dimeglumine (Magnevist, Bayer Schering Pharma AG) or gadobenate dimeglumine (Multihance, BD), administered at a dose of 0.1 mmol/kg. Imaging parameters: The MR imaging protocol was as follows: T1-weighted images were acquired using an echo time (TE) 2.46ms, a repetition time (TR) 200ms, and voxel size 0.9mm × 0.7mm × 5mm. T2-weighted FLAIR images were acquired using an inversion time of 2500ms, TR 9000ms, TE 93ms, and voxel size of 0.9mm × 0.9mm ×5mm.
Area of acquisition	Whole-brain

Diffusion MRI ☐ Used ☒ Not used

Preprocessing

Preprocessing software	No preprocessing performed, MR images were directly analyzed in the hospital PACS.
Normalization	No normalization used
Normalization template	No template used
Noise and artifact removal	None used
Volume censoring	No volume censoring was performed

Statistical modeling & inference

Model type and settings	No model used
Effect(s) tested	n/a

Specify type of analysis: ☒ Whole brain ☐ ROI-based ☐ Both

Statistic type for inference (See Eklund et al. 2016)	n/a
Correction	Multiple comparisons were not performed

Models & analysis

n/a	Involved in the study
☒	☐ Functional and/or effective connectivity
☒	☐ Graph analysis
☒	☐ Multivariate modeling or predictive analysis
